# Recent advances in ultrasound microbubble-mediated gold-based nanomaterials for cancer theranostics

**DOI:** 10.3389/fchem.2026.1899856

**Published:** 2026-07-31

**Authors:** Xinyi Ma, Xiaobing Chen, He Li, Jikang Gao

**Affiliations:** Department of Ultrasound, The First People’s Hospital of Linping District, Hangzhou, China

**Keywords:** cancer, gold-based nanomaterials, theranostics, tumor, ultrasound microbubbles

## Abstract

In the realm of oncology, precision cancer diagnosis and treatment is a current research emphasis and development trend. Its main objective is to accomplish closed-loop management of “early diagnosis - precise treatment - real-time monitoring” in order to increase the effectiveness of diagnosis and treatment while lowering toxic and adverse therapeutic effects. Because of their special optical, electrical, and biocompatible benefits, gold-based nanomaterials have demonstrated wide application prospects in tumor imaging and treatment. Ultrasound microbubbles (UMBs) are a novel form of ultrasonic contrast agent and drug delivery vehicle with superior biocompatibility, ultrasonic responsiveness, and targeted modification potential. As powerful delivery vehicles, UMBs play an increasingly vital role in clinical theranostics by efficiently mediating the targeted delivery, controlled release, and synergistic therapeutic effects of gold-based nanomaterials. The varieties of UMBs, the categorization of gold-based nanomaterials, and the advancements in research on their applications in common malignancies are the main topics of this mini-review. Lastly, it provides pertinent references for the study and clinical applications of UMB-mediated gold-based nanomaterials in cancer diagnosis and treatment. It also briefly examines the present research prospects and obstacles and looks forward to future development paths.

## 1 Introduction

Despite continuous advances in multimodal imaging and anti-tumor therapeutic modalities, substantial clinical bottlenecks persist in tumor management (Chen et al., 2026; Santiago-Sánchez et al., 2024; Thomas, 1983). Conventional imaging modalities fail to enable early detection of tiny primary lesions and micrometastases; meanwhile, systemic radiotherapy and chemotherapy lack tumor-targeting specificity, frequently inducing severe off-target toxicities and sustaining high postoperative recurrence rates among patients (Chidambaram et al., 2011; Zafar et al., 2025). Defined as an integrated nanomedical strategy that unites tumor diagnosis and targeted therapy, theranostics facilitates lesion localization, controlled drug delivery, and real-time therapeutic response monitoring, rendering it a core paradigm to resolve the aforementioned clinical dilemmas (Kashyap et al., 2023). Owing to their distinctive physicochemical properties, gold-based nanomaterials stand out as preferred building blocks for theranostic platforms and have emerged as a central research Frontier in oncology. Their hallmark merit originates from the localized surface plasmon resonance (LSPR) effect: upon irradiation at specific wavelengths, these nanostructures generate intense scattering and absorbance signals, laying a solid foundation for multimodal imaging (Eker et al., 2024; Theodorou et al., 2023). Gold-based nanomaterials integrate multiple imaging modalities, including photoacoustic, computed tomography (CT), and Raman imaging. Such synergistic imaging capabilities enable precise characterization of tumor size, morphology, location, and invasive boundary, thereby improving diagnostic accuracy (Barlow and Kim, 2025; Fernandes, 2024; Li et al., 2022). Additionally, these nanomaterials exhibit favorable biocompatibility and readily undergo surface functionalization. By conjugating functional moieties, including targeting ligands, chemotherapeutic agents, and photosensitizers, they integrate multiple therapeutic modalities, such as photothermal therapy (PTT), photodynamic therapy (PDT), and targeted drug delivery, realizing synergistic theranostic performance characterized by image-guided intervention and real-time therapeutic feedback (Damani et al., 2024; Fernandes, 2024). Nevertheless, gold nanocarriers exhibit inherent drawbacks in vivo delivery. They are readily captured by the mononuclear phagocyte system, leading to insufficient tumor accumulation and uncontrolled drug release, which greatly restricts their clinical transformation (Bano et al., 2025; Jackson et al., 2025).

Ultrasound microbubbles (UMBs) refer to micron-sized vesicular structures composed of a gaseous core and an outer shell. The shell can be fabricated from biocompatible raw materials, including lipids, polymers, and proteins, while the interior is filled with inert gases, such as air, and perfluoropropane (Navarro-Becerra and Borden, 2023; Rudakovskaya et al., 2022; Zhang et al., 2025). With the interdisciplinary progress of nanomedicine and ultrasound medicine, the drug delivery and media-mediated functions of microbubbles (MBs) have been extensively investigated, and MBs serve as core carriers for boosting the *in vivo* delivery efficiency of nanomaterials (Chapla et al., 2022; Lu et al., 2022). Featuring prominent biocompatibility, UMBs can compensate for the inherent drawbacks of free gold nanomaterials. Benefiting from ultrasound-triggered cavitation effects and their modifiable outer surfaces, UMBs prolong the blood circulation time of gold-based nanostructures, enhance tumor penetration capacity via sonoporation, and permit spatiotemporally controllable drug release (Clements and Griffiths, 1987).

**FIGURE 1:**
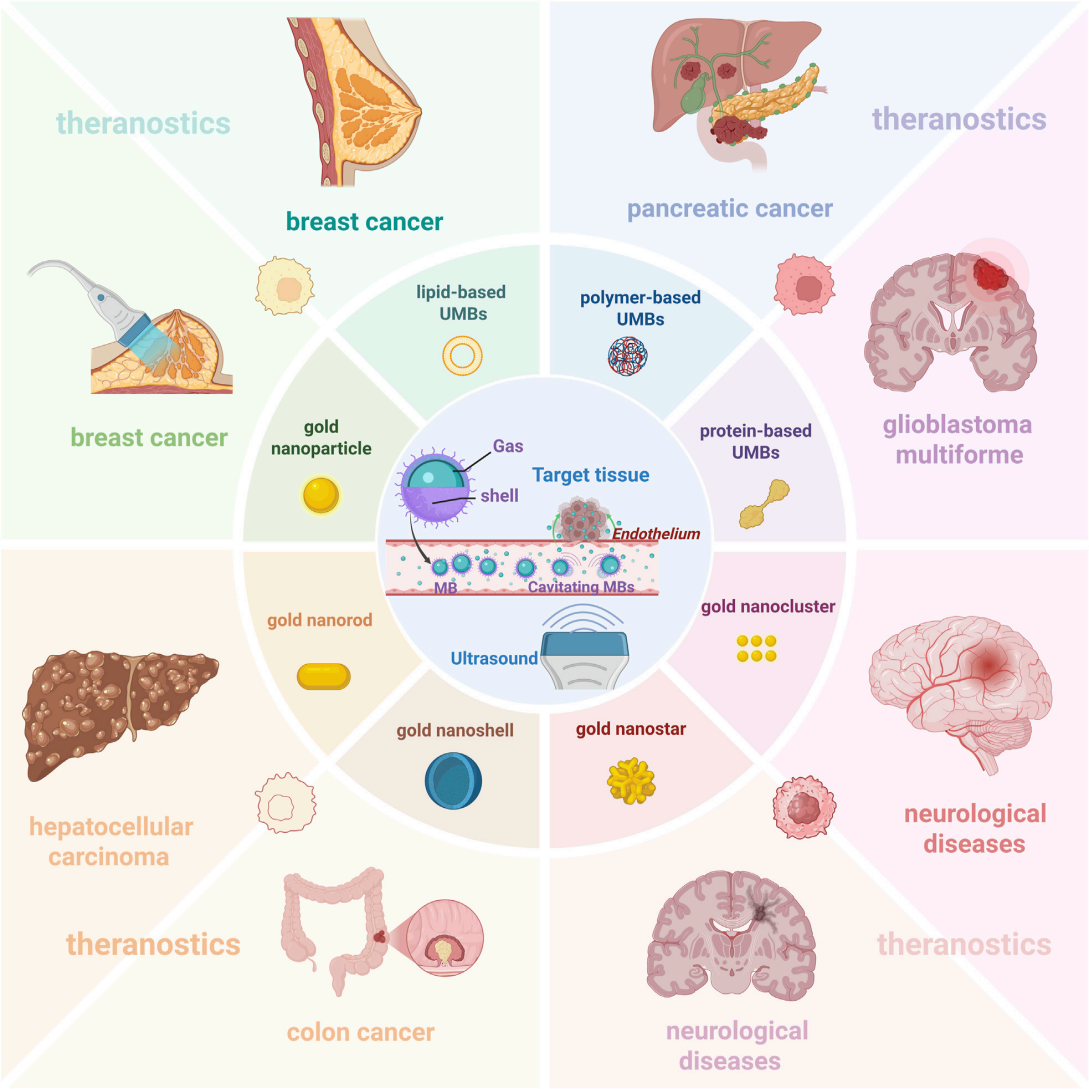
Ultrasound microbubble-mediated gold-based nanomaterials for cancer theranostics. It was created by BioRender.

At present, numerous independent experiments have sufficiently verified the translational potential of diverse UMB-gold nanocomposite theranostic platforms for tumor management (Barmin et al., 2021; Tarapacki and Karshafian, 2015). However, systematic review articles covering this field remain scarce, leaving two critical research gaps that severely hinder comprehensive understanding of such composite systems and their multi-scenario clinical translation. First, gold nanomaterials encompass multiple morphologies, including gold nanoparticles (AuNPs), gold nanorods (AuNRs), gold nanoshells (AuNSs), gold nanostars (AuNSTs), and gold nanoclusters (AuNCs). The regulatory patterns by which different conjugation modes modulate the ultrasonic responsiveness, *in vivo* circulation stability, and drug release kinetics of composite platforms remain ambiguous, and no unified structure–activity relationship has been summarized to date. Second, current research predominantly focuses on single tumor models, lacking cross-sectional comparative analyses of breast cancer, digestive system tumors, and central nervous system (CNS) tumors. This review constructs a multi-dimensional analytical framework centered on material classification and practical disease applications. It first categorically introduces three mainstream shell materials for MBs and five typical gold nanomaterials. Afterwards, the latest research advances of these nanocomposite systems in breast, digestive, and CNS tumors are summarized (Figure 1). Furthermore, this work deeply dissects fundamental core bottlenecks restricting clinical translation and proposes innovative prospective research directions, delivering feasible strategies for carrier structural optimization and clinical implementation.

## 2 Types of UMBs

UMBs are a class of micron-sized bubbles made of a shell material and a core gas. They can be classified using a variety of methods, the most typical of which is based on the composition of the shell material (Moosavifar et al., 2024). Lipid-based UMBs, polymer-based UMBs, and protein-based UMBs are now the three types of UMBs that are most frequently utilized in fundamental research and clinical translation (Lee et al., 2017; Xiong et al., 2011).

### 2.1 Lipid-based UMBs

The prominent and well-established kind of ultrasound contrast and delivery vehicles are liposomal UMBs. Their shell is mainly made up of natural lipids (like phosphatidylcholine and phosphatidylethanolamine) or synthetic lipid analogues (Lu et al., 2025); their inner core often contains inert gases like perfluoropropane and perfluorobutane (Martin and Dayton, 2013). These MBs are rapidly metabolized *in vivo*, display exceptional biocompatibility and biodegradability, and rarely cause accumulated toxicity. In the interim, the lipid shell is easily adaptable and capable of functional modification. It is capable of loading gold-based nanomaterials through internal encapsulation or surface conjugation, in addition to achieving active targeted accumulation by conjugating targeting ligands such as antibodies and peptides (Duan et al., 2020).

### 2.2 Polymer-based UMBs

The shell of polymer-based UMBs is mainly composed of highly biocompatible polymeric materials, including common types such as poly (lactic-co-glycolic acid) (PLGA), chitosan, and polyethylene glycol (PEG) (Ghamkhari et al., 2023). The principal advantages of polymer-based MBs are their substantially improved structural stability, significantly extended *in vivo* circulation half-life, and expanded loading capacity for gold-based nanomaterials. Furthermore, by adjusting the molecular weight, molecular structure, and polymerization degree of the polymer, their ultrasound-responsive properties and *in vivo* metabolism rate can be flexibly optimized to further meet the requirements of different theranostic scenarios (Hark et al., 2024).

### 2.3 Protein-based UMBs

Protein-based UMBs use natural proteins (such as albumin, hemoglobin, collagen, etc.) as the main components of their shell (Dhara et al., 2024). These MBs are biosafe and show an exceptionally high level of biocompatibility, and they rarely elicit immune responses in the body. Simultaneously, protein molecules can establish stable bonds with a variety of functional substances by utilizing their distinctive molecular structures, which leads to a high loading efficiency. In addition, their protein shell can be completely degraded by enzymatic hydrolysis *in vivo* without residual toxicity, showing favorable *in vivo* metabolic properties.

In summary, various UMBs possess distinct advantages and drawbacks with considerable discrepancies in their physicochemical and functional performances. Specifically, lipid-based UMBs show superior biocompatibility yet suffer from fragile structural integrity and a short *in vivo* circulation half-life. Polymer-based UMBs feature outstanding mechanical robustness and structural stability, whereas they display inferior sensitivity to ultrasonic stimulation. Protein-based UMBs are equipped with abundant surface modification sites that facilitate the immobilization of functional molecules, but their large-scale manufacturing remains a major challenge. Benefiting from the unique material merits of each subtype, the three categories of MBs can efficiently carry gold-based nanomaterials to achieve targeted *in vivo* delivery, which renders them suitable for diverse tumor theranostic scenarios.

## 3 Classification of gold nanomaterials

Gold-based nanomaterials are a functional nanomaterial system that is composed of gold as the main part. Pure gold nanomaterials are the fundamental and central units of gold-based nanomaterials, and their classification is primarily determined by their size and morphology (Venditti, 2019). AuNPs, AuNRs, AuNSs, AuNSTs, and AuNCs are currently the most extensively studied and applied in tumor theranostics.

### 3.1 AuNPs and AuNRs

AuNPs are the most frequently employed gold nanomaterials. They comprise a spherical or subspherical morphology, as well as a flexible surface modification and favorable biocompatibility. They can be employed as CT contrast agents or to obtain highly sensitive tumor imaging through surface-enhanced Raman scattering. Dual-modal imaging systems can be established to enhance localization accuracy by combining them with UMBs (Shakeri-Zadeh et al., 2021). Additionally, they can carry medications or use PTT effects to destroy tumor cells for therapeutic purposes. The rod-like structure of AuNRs confers a strong LSPR effect and high photothermal conversion efficiency, making them excellent carriers for tumor PTT (Yim et al., 2022). They can precisely target tumors under imaging guidance to optimize therapeutic efficacy, reduce damage to normal tissues caused by near-infrared light, and improve treatment safety (Gao et al., 2024).

### 3.2 AuNSs, AuNSTs, and AuNCs

AuNSs feature a core–shell structure, typically composed of a silica or polystyrene core coated with an ultrathin gold layer (Rastinehad et al., 2019). For imaging applications, they can act as dual-modal CT and photoacoustic contrast agents to enhance imaging contrast and enable precise tumor localization (Chen et al., 2023). In therapeutic applications, they generate a strong photothermal effect under near-infrared irradiation to ablate tumors, and their easily modifiable surface allows drug loading for the construction of synergistic therapeutic systems (Chen et al., 2025; Nouri et al., 2019). AuNSTs have a controlled three-dimensional star-shaped structure with powerful electric field amplification at their sharp tips and extraordinary LSPR (Liu et al., 2023). In the meantime, targeting molecules, chemotherapeutic drugs, and other functional cargos can be easily modified and loaded thanks to their many surface-active locations. As a result, AuNSTs have a wide range of potential applications, including effective PTT and multimodal imaging-guided tumor localization (Hernández Montoto et al., 2018; Liu et al., 2015). As an ultrasmall gold-based nanomaterial with a size close to the Fermi wavelength, AuNCs possess unique molecular-like fluorescence properties and excellent biosafety, thus exhibiting distinct advantages in tumor fluorescence imaging, targeted tracing, and precision therapy (Cui et al., 2023).

Combined with the coupling characteristics of the aforementioned five types of gold-based nanomaterials and UMBs, matching the type of MB carrier with the morphology of gold nanomaterials enables the fabrication of integrated nanotheranostic platforms that integrate precise diagnosis via multimodal imaging and site-specific targeted therapy. To further elaborate on the clinical translational potential and practical performance of this technology, the following section systematically summarizes the latest research advances of such integrated theranostic platforms, focusing on three prevalent malignant tumors, namely breast cancer, digestive system malignancies, and CNS tumors (Table 1).

**TABLE 1:** Applications of ultrasound microbubble-mediated gold-based nanomaterials in various diseases.

Types of gold nanomaterials	Disease	System	Core viewpoints	Diagnostic/therapeutic methods	References
gold nanoparticle	breast cancer	mammary system	smart gold nanoparticle-stabilized microbubbles realize tumor ultrasound/photoacoustic dual-modal imaging, and ultrasound sonoporation boosts intratumoral gold nanoparticle accumulation for photothermal therapy	ultrasound/photoacoustic dual-mode imaging; photothermal therapy	Yoon et al. (2018)
gold nanoshell	breast cancer	mammary system	Her-2-targeted PLGA liquid fluorocarbon microcapsules coated with gold nanoshells achieve targeted breast tumor ultrasound imaging and photothermal ablation *in vitro*	ultrasound imaging; photothermal therapy	Zhang et al. (2018)
gold nanorod	breast cancer	mammary system	VEGFR2-targeted microbubbles encapsulating gold nanorods rely on acoustic cavitation-induced sonoporation to synergistically improve intracellular delivery of gold nanorods, amplifying plasmonic photothermal therapy efficacy	ultrasound/photoacoustic dual-mode imaging; photothermal therapy	Wang et al. (2014)
gold nanoparticle	breast cancer	mammary system	ultrasound-microbubble-mediated sonoporation facilitates cellular uptake of PEGylated gold nanoparticles to synergistically enhance breast cancer laser photothermal cytotoxicity	photothermal therapy	Tarapacki and Karshafian (2015)
gold nanoparticle	pancreatic cancer	digestive system	ultrasound-targeted microbubble destruction (UTMD) promotes dendrimer-entrapped gold nanoparticle co-delivery of gemcitabine and miR-21 inhibitor for combined chemo-gene therapy against pancreatic cancer	chemo-gene combination therapy	Li et al. (2018)
gold nanoparticle	hepatocellular carcinoma	digestive system	microbubble-mediated sonoporation elevates intracellular gold nanoparticle concentration in hepatocellular carcinoma cells, inducing amplified DNA damage and enhancing megavoltage X-ray radiosensitization effects	radiosensitization enhancement therapy	Lu et al. (2020)
gold nanorod	colon cancer	digestive system	multifunctional microbubble carriers deliver gold nanorods for colon tumor ultrasound molecular imaging, photoacoustic visualization, and synergistic photothermal tumor ablation	ultrasound molecular imaging/photoacoustic imaging; photothermal therapy	Wang et al. (2014)
gold nanorod	neurological diseases	central nervous system	microbubble-assisted focused ultrasound mediates transcranial gold nanorod delivery, enabling *in vivo* brain molecular photoacoustic imaging of central nervous lesions	photoacoustic imaging	Hartman et al. (2019)
gold nanocluster	neurological diseases	central nervous system	focused ultrasound-microbubble combined intranasal delivery bypasses the blood-brain barrier to transport radiolabeled gold nanoclusters, supporting multimodal PET/fluorescence brain molecular imaging	PET/CT imaging; fluorescence imaging	Ye et al. (2018)
gold nanoparticle	glioblastoma multiforme	central nervous system	MRI-guided focused ultrasound transiently disrupts the blood-brain barrier to safely and locally augment parenchymal gold nanoparticle infiltration in glioblastoma for subsequent therapeutic intervention	MRI-guided FUS/CT	Etame et al. (2012)

## 4 Recent research progress in common diseases

### 4.1 Research progress in breast diseases

Breast cancer is the most prevalent malignant tumor in women. Its main treatments include surgery, chemotherapy, and radiotherapy; however, the postoperative recurrence rate is relatively high, and the toxic side effects of chemotherapeutic drugs severely affect patients’ quality of life (Cai et al., 2025; GBD, 2023 Cancer Collaborators, 2025). Recently, numerous research projects have linked gold-based nanomaterials with UMBs to create various unique nanoplatforms for breast cancer diagnosis and theranostics, opening up new avenues for the disease’s evaluation.

A previous study (Yoon et al., 2018) fabricated a AuNP-stabilized intelligent UMB system, which exhibited superior integrated theranostic performance. It was demonstrated that this system permitted effective ultrasound contrast imaging and ultrasound/photoacoustic dual-modal imaging guidance in a mouse breast cancer model. Furthermore, under ultrasound triggering, the system could promote the accumulation of AuNPs at tumor sites via sonoporation and generate a pronounced PTT effect under near-infrared light irradiation, thereby effectively inhibiting tumor growth. Collectively, these results revealed favorable biosafety and promising potential for precise theranostics. To realize precise targeted therapy for patients with HER2-positive breast cancer, several studies have developed UMB-based theranostic platforms endowed with specific targeting functions. In this context, Zhang et al. (2018) constructed Her2-PFOB@PLGA@Au UMBs with uniform size and favorable biocompatibility. Such a system not only allowed enhanced ultrasound imaging but also produced a remarkable PTT effect upon near-infrared laser irradiation, thus permitting specific targeting and effective killing of HER2-positive breast cancer cells. This work offers a new insight into the targeted theranostics of breast cancer. Furthermore, multifunctional combinatorial theranostic platforms have been engineered according to tumor-specific targets, further broadening the applicability of such systems. A relevant study (Wang et al., 2014) developed VEGFR2-targeted albumin-based multifunctional MBs incorporating AuNRs on the shell layer. Upon ultrasound exposure, cavitation and sonoporation effects markedly promoted the enrichment and intracellular internalization of AuNRs in breast cancer, thus strengthening the plasma-mediated PTT ablation effect. Simultaneously, ultrasound/photoacoustic dual-modal imaging was realized, offering a promising strategy for precision tumor theranostics. Moreover, for triple-negative breast cancer (TNBC), which is characterized by poor clinical prognosis and limited effective therapeutic targets, a study demonstrated that PEGylated AuNPs combined with UMBs also exerted significant synergistic therapeutic efficacy in the MDA-MB-231 breast cancer cell model. UMBs enhanced nanoparticle delivery via sonoporation, hence markedly strengthening near-infrared laser-mediated PTT ablation. These results provide robust experimental evidence for improving the phototherapeutic efficacy against triple-negative breast cancer (Tarapacki and Karshafian, 2015). Collectively, the above-mentioned studies have verified that combinatorial systems consisting of UMBs and gold-based nanomaterials can effectively achieve precise imaging, targeted PTT, and chemosensitization against breast cancer, offering an innovative strategy for individualized theranostics of breast malignancies.

### 4.2 Research progress in the digestive system

At present, studies on the combined system of UMBs and gold-based nanomaterials in digestive system tumors mainly focus on pancreatic cancer (PC), hepatocellular carcinoma (HCC), and colon cancer (CC).

In the context of PC therapy, accumulating investigations have revealed encouraging therapeutic potential and promising clinical prospects for the combined application of UMBs and gold-based nanomaterials. In a representative study, dendrimer-encapsulated AuNPs were employed as a versatile delivery platform for the co-loading of gemcitabine and miR-21 inhibitors in conjunction with ultrasound-targeted microbubble destruction (UTMD) for PC treatment (Li et al., 2018). Findings from this research demonstrated that UTMD markedly promoted cellular internalization of therapeutic agents and improved their accumulation at tumor sites. By downregulating miR-21 expression, this nanoplatform effectively reversed chemoresistance in PC, enabling synergistic enhancement of chemotherapy and gene therapy. Furthermore, it significantly suppressed the proliferation of PC cells, thereby establishing a novel and highly efficient delivery approach for the targeted combinatorial therapy of PC. Similarly, this combined strategy shows significant application value in the field of radiosensitization for HCC. A study by Lu et al. (2020) elucidated that AuNPs-loaded UMBs combined with ultrasound-mediated sonoporation can markedly facilitate the delivery and accumulation of gold-based nanomaterials in human HCC Huh7 cells. This strategy effectively enhances the cytotoxic effect of megavoltage X-rays on HCC cells, aggravates DNA damage, and suppresses cell survival, thus offering a novel synergistic therapeutic paradigm for radiosensitization in HCC. Moreover, this theranostic platform has also been successfully extended in the precise treatment of CC. A study by Wang et al. (2014) demonstrated that in the CT26 CC model, the VEGFR2-targeted AuNR-MB complex could specifically localize in tumor neovascularization and achieve long-term retention, laying a foundation for the effective accumulation of AuNRs in tumor tissues. Furthermore, UTMD enhances the intratumoral delivery of AuNRs, and the combined near-infrared irradiation achieves effective plasmonic PTT, providing a feasible strategy for CC theranostics.

### 4.3 Research progress in the CNS

The diagnosis and treatment of CNS diseases are often limited by the obstruction of the blood-brain barrier (BBB), making it difficult for nanodrugs and imaging probes to effectively enter the brain and exert their functions. Relevant studies have been carried out from the perspectives of BBB opening, intranasal targeted delivery, and magnetic resonance (MR)-guided precision, gradually achieving efficient accumulation and safe delivery of gold nanomaterials in brain tissues, which provides a new idea for the imaging diagnosis and targeted therapy of brain diseases.

A study has adopted UMBs combined with focused ultrasound (FUS) technology to reversibly open the BBB, facilitating efficient delivery of AuNRs into the brain (Hartman et al., 2019). In this research, AuNRs were used as contrast agents, and photoacoustic imaging was employed for *in vivo* tracing of their distribution in the brain. The results verified the feasibility of this combined strategy for *in vivo* molecular neuroimaging, providing a new idea for the delivery of intracerebral nanoprobes and noninvasive imaging. In addition to the conventional approaches for BBB opening, intranasal delivery offers a more noninvasive, innovative strategy for the penetration of nanomaterials into the brain. A study by Ye et al. (2018) established a FUS combined with MB-mediated intranasal drug delivery system, enabling efficient and targeted delivery of AuNCs to brain tissues. This strategy capitalizes on the synergistic effect of ultrasound and MBs to enhance the enrichment of gold-based nanomaterials in the brain, furnishing a noninvasive, targeted, safe, and efficient novel approach for the imaging diagnosis and drug delivery of brain diseases. This extends the application of nanomaterials in the field of the CNS and lays a delivery foundation for targeted imaging/therapy of brain tumors. In terms of precise delivery and biosafety, MR-guided technology has further promoted the development of this field. A study adopted MR-guided FUS combined with lipid UMBs, utilizing the ultrasound-induced cavitation effect of MBs to reversibly open the BBB, successfully achieving efficient delivery of PEGylated AuNPs into the cerebral parenchyma of rats (Etame et al., 2012). The results showed that this combined intervention could significantly improve the enrichment level of AuNPs in brain tissue, with mild and reversible damage to brain tissue, indicating good biosafety. This strategy effectively overcomes the BBB restriction on nanoparticle delivery, establishes a reliable methodological basis for brain tumor theranostics using gold-based nanomaterials, and provides valuable insights for subsequent brain tumor research.

Spherical AuNPs attach to lipid or albumin MBs via physical adsorption and electrostatic attraction (Yoon et al., 2018). In contrast, anisotropic porous gold nanostructures, including AuNRs and AuNSs, integrate with MBs by covalent conjugation or *in-situ* growth (Wang et al., 2014). Physically assembled MBs retain intact hollow frameworks and deliver favorable cavitation and imaging performance, despite fragile binding forces. Covalent treatment increases MB rigidity and attenuates cavitation, yet anisotropic gold nanostructures offset this limitation by amplifying photoacoustic signals for multimodal imaging (Chapla et al., 2022). Once in circulation, non-covalent complexes readily shed gold components via competitive displacement by plasma proteins. Covalently linked composites withstand blood shear stress with minimal dissociation, making them ideal for BBB-crossing long-circulating delivery (Barmin et al., 2021). Non-covalent carriers solely encapsulate drugs in inner cavities and produce abrupt drug release upon ultrasound stimulation. In comparison, covalent platforms support dual-site drug loading. Adjusted ultrasound power enables sequential controlled release to synergize PTT, chemotherapy, and gene therapy.

## 5 Conclusion

With continuous advances in nanoscale synthesis and ultrasound technology, the combined system of gold-based nanomaterials mediated by UMBs has provided a more efficient and safe novel strategy for cancer theranostics. However, significant challenges still exist in the current system. Firstly, there are certain limitations in carrier performance: lipid-based MBs exhibit relatively insufficient stability and a short circulation period (Al-Jawadi and Thakur, 2020); polymer-based MBs lack sensitivity to ultrasound response (Moosavifar et al., 2024); protein-based MBs are difficult to scale up for mass production (Rudakovskaya et al., 2022); and the composite stability between various types of MBs and gold-based nanomaterials is poor, which is prone to dissociation in the body and thus affects delivery efficiency. Secondly, the targeting and enrichment effects are still insufficient. Single-target modification fails to adequately adapt to the heterogeneous characteristics of tumors, resulting in a relatively low enrichment rate at tumor sites (Gao et al., 2021; McCarrick et al., 2021). Meanwhile, the ultrasonic cavitation effect is prone to cause mild damage to normal vascular endothelium (Hwang et al., 2006). In addition, numerous technical hurdles constrain the utility of this composite platform. Researchers have yet to thoroughly clarify long-term *in vivo* metabolic patterns and biocompatibility of gold-based nanomaterials. Once circulated in blood, these particles develop a protein corona that masks surface targeting motifs and hinders tumor accumulation. Scarce systematic research addresses dose-effect correlations tied to bodily material deposition. Optical scattering across biological tissues weakens synergistic therapeutic outcomes, producing inconsistent and poorly reproducible treatment results. These prominent barriers greatly slow clinical translation, which necessitates prompt modification of the composite architecture (Jakic et al., 2024; Kus-Liśkiewicz et al., 2021). Apart from the above material and biological drawbacks, scalable fabrication, device standardization, and clinical compatibility block this composite system’s translation. AuNPs liquid synthesis produces severe batch variations with inconsistent particle sizes and optical traits; scaled-up cost assessments and economic comparisons with commercial MBs remain absent. Divergent ultrasound parameters across devices shift the system’s cavitation threshold and degrade outcome reproducibility without universal calibration standards. Moreover, the theranostic platform fails to match routine ultrasound workflows or integrate smoothly with multimodal tumor biopsy and treatment, requiring systematic follow-up research to resolve these translational hurdles.

Future research should address translational obstacles from material, engineering, and clinical perspectives. On one hand, researchers shall optimize the structure of composite MBs and multi-target modification systems. These improvements will enhance carrier stability and intratumoral accumulation. Comprehensive evaluation frameworks for the metabolism and biosafety of gold-based nanomaterials also need to be established (Chen et al., 2019; Wang et al., 2019); on the other hand, translational engineering bottlenecks require resolution. Key issues include batch-to-batch inconsistency during large-scale production, inconsistent parameters across ultrasonic devices, and limited compatibility with standard clinical workflows. Two cutting-edge research directions can be further explored. First, ultrasonic microbubble cavitation can reversibly adjust tumor vascular permeability. This facilitates deeper penetration and prolonged retention of nanomaterials within tiny lesions. The signal-to-noise ratio of photoacoustic/ultrasound dual-modal imaging for primary early lesions and micrometastases can thus be elevated, enabling higher detection rates of minute tumors (Theek et al., 2016). Second, an integrated platform combining MBs, gold nanomaterials, and immune checkpoint inhibitors can be constructed. Ultrasound-triggered localized and controllable drug release remodels the immunosuppressive tumor microenvironment *in situ*. This platform activates systemic anti-tumor immunity and achieves long-term suppression of distant tumor metastasis and postoperative recurrence (Liu et al., 2018). The above two strategies center on early precise diagnosis and immunological radical therapy, respectively. They deliver innovative research avenues to advance the clinical translation of this composite system. Additionally, unified preparation standards and therapeutic parameters, as well as clinical evaluation systems, should be established. Furthermore, efforts should be made to promote in-depth connection between basic research and clinical application in order to fully exert its clinical value in precise cancer diagnosis and treatment.
